# The role of emotional quotients and adversity quotients in career success

**DOI:** 10.3389/fpsyg.2023.1128773

**Published:** 2023-02-09

**Authors:** Yuyang Zhao, Biao Sang

**Affiliations:** ^1^Department of Social Work, School of Sociology and Political Science, Shanghai University, Shanghai, China; ^2^Lab for Educational Big Data and Policymaking, Shanghai Academy of Educational Sciences, Shanghai, China

**Keywords:** trait emotional quotient, adversity quotient, career success, organizational commitment, job position

## Abstract

Career success has been considered equally important for both personal and organizational development. The purpose of the current study was to examine how trait emotional quotient (EQ) and adversity quotient (AQ) contribute to individuals’ objective career success (job position) and subjective career success (organizational commitment). Participants included 256 Chinese adults who completed four measurements—the Self-Reported Emotional Intelligence Test, Resilience Scale, Grit Scale, and the Affective, Continuance, and Normative Commitment Scale—and provided demographic information. After validating the four scales used in this study, multiple regression analysis revealed that only one aspect of trait EQ (regulation of emotion) positively predicted one component of organizational commitment (affective commitment). Adversity quotient was measured on two dimensions: resilience and grit. Only consistency of interest (grit) positively predicted affective commitment. Perseverance of effort (grit) and acceptance of self and life (resilience) positively predicted normative commitment. Personal competence (resilience) positively predicted continuance commitment but negatively predicted normative commitment. Only acceptance of self and life (resilience) positively predicted job position. Overall, these findings demonstrate the specific influence of trait EQ and AQ on career success for organizational professionals who want to improve organizational productivity as well as individuals who want to achieve success at work.

## Introduction

As early as the 1970s, career success has already been intensively studied in the fields of management and applied psychology (Ng et al., 2005; Sullivan and Baruch, 2009). Personal career success is not only vital for individuals but also for organizations, as employees’ career success will eventually contribute to an organization’s success (Judge et al., 1999). With the development of the world as well as the rapid change of the global economic structure around the world, researchers need to continue to explore the potential factors that contributed to individuals’ career success, which is equally important for both individual and organizational growth. Career success is often referred to as an outcome of an individual’s career experience (Seibert et al., 1999; Arthur et al., 2005).

Even though scholars indicated that little attention has been devoted to discussing the nature of career success (e.g., Sturges, 1999; Greenhaus, 2003; Heslin, 2003, 2005), various theories (e.g., Chartrand and Rose, 1996; Seibert et al., 2001) and models (e.g., Holland, 1997) were discuses by previous researchers. One of the most cited frameworks for operationalized career success was Hughes’ (1937, 1958) study, which made a theoretical distinction between objective career success (OCS) and subjective career success (SCS). Specifically, Hughes operationalized objective career as the experienced that is directly observable, measurable, and verifiable by an impartial third party, while the subjective career is only experienced by the person engaged in the individual’s career. Thus, OCS is often defined as outcomes that can be directly observed and measured in a standardized way (Hughes, 1937, 1958; Arthur et al., 2005), which was also referred to as extrinsic career success in some other studies (Judge et al., 1999; Erdogan et al., 2004). These outcomes included salary, a person’s organizational position, promotion history, and occupational prestige (Van Maanen, 1977; Dries et al., 2009), which have been considered the features of career success across different societies (Nicholson, 2000). Even though previous studies often employed individuals’ salaries and promotions to represent OCS, the current research used job positions based on two main reasons. On the one hand, individuals received higher salaries and promotions do not necessarily make people feel proud or successful (Schein, 1978; Korman et al., 1981). Heslin (2005) suggested that researchers should “conceptualize and assess objective success in a manner that is guided by the career concerns and status hierarchies” (p. 116), which can be represented by job position. On the other hand, the salary was not stable and often fluctuated according to geographic location. For example, average annual salaries in more developed areas (i.e., New York) are higher than in less developed areas (i.e., Alaska; U.S. Bureau of Labor Statistic, 2020). In addition, the likelihood of receiving promotions largely depends on the type of organization and profession, and such concerns are often magnified when collecting national data or collecting data on the internet. In contrast, the job position is more objective and standardized. Most people know what manager means and what to expect from individuals at a managerial level. Therefore, the job position was selected to represent OCS in the current study.

As discussed by Shockley et al. (2016), career success should not only be indicated by objective factors, but also by fewer tangible factors that required subjective interpretation, such as SCS which was also called intrinsic career success (Judge et al., 1999; Erdogan et al., 2004). Subjective career success is generally defined by using more personally meaningful career outcomes (Shockley et al., 2016) or an individual’s personal reaction to their unfolding career experiences (Hughes, 1937, 1958), such as job or career satisfaction (Thorndike, 1934; Kirchmeyer, 2006; Abele and Spurk, 2009). Even though job or career satisfaction is often used as a proxy for SCS (Tsui and Gutek, 1984; Judge et al., 1995; Boudreau et al., 2001), the organizational commitment was selected to represent SCS in the current study for two main reasons. On the one hand, researchers discussed four major concerns regarding the use of job satisfaction to represent SCS and indicated that SCS should include individuals’ reactions to “actual and anticipated career-related attainments” (Heslin, 2005, p. 117), such as a sense of identity (Law et al., 2002) and organizational commitment (Cabrera-Suárez and Martín-Santana, 2012). On the other hand, individuals’ organizational commitments indicate a deeper psychological connection with the organization or the career than job satisfaction. Meyer and Allen (1991) defined organizational commitment as “a psychological state that (a) characterizes the employee’s relationship with the organization, and (b) has implications for the decision to continue membership in the organization” (p. 67). Organizational commitment is a relational psychological contract with the organization or career, which is different from other contracts since it is based on employees’ perceptions rather than actual reality. Employees can be satisfied with their organization but not work hard and achieve success in their job. However, if individuals are committed to their organization, such commitment may encourage or foster individuals to continually investigate energy and time into their careers to obtain success. Therefore, in the current study, career success was measured by both OCS, as represented by an individual’s position in the organization, and SCS, as represented by organizational commitment.

Under current global economic circumstances, work environments have become more flux and complex than ever. Apart from controllable influences, which are supported by the life-span perspective on career development (Vondracek et al., 1986), no one can anticipate the impact of new technologies or economic instability on one’s career. To face these uncontrollable changes and situations, individuals need to master stress-coping skills associated with career uncertainties and unanticipated changes, as well as become more effective and efficient at their jobs, which requires high levels of cognitive and emotional adjustment skills (Coetzee and Harry, 2014). Regarding cognitive adjustment skills, over the past few decades, meta-analyses have found individuals’ general mental ability (i.e., *g*) to be a predictor of job performance and success (Hunter, 1986; Schmidt and Hunter, 1998; Kuncel et al., 2004). However, more studies have shown that IQ has little predictive power for job performance and it has less predictive power than it once did (i.e., Jencks, 1998), which might be due to the “Flynn effect” (Flynn, 2007). Moreover, the correlation between IQ and job performance also shows cultural differences. For example, one study found this correlation to be weaker in China and the Middle East than in the United States and Europe (Byington and Felps, 2010).

Due to the concern about the predictive power of cognitive ability (i.e., general *g*) on job performance, increasing studies have turned their attention to the effects of non-cognitive factors. For example, Goleman (2000) pointed out that, across all job categories, emotional competence was twice as important as IQ, since most of the abilities important for effective performance were related to emotional competence. Goleman further indicated that emotional competence that was used to facilitate individual performance can be represented as an emotional quotient (EQ). Besides the influence of emotional competencies, how individuals deal with adversity in their life and work is often discussed by researchers, especially in positive psychology (Seligman, 2011; Southwick et al., 2014). Stoltz (1997) identified the concept of adversity quotient (AQ), which try to address questions such as why people with a high IQ and EQ can still fall short of their potential. Therefore, the current study suggested that EQ and AQ play a vital role in how individuals deal with emotional, interpersonal, and occupational adversities and obstacles.

## Theoretical framework

### Emotional quotient

There are two main types of EQ: ability EQ and trait EQ. Ability EQ was originally defined as “the subset of social intelligence that involves the ability to monitor one’s own and other’s feelings and emotions, to discriminate among them, and to use this information to guide one’s thinking and actions” (Salovey and Mayer, 1990, p. 189). They further identified a threefold framework for EQ: appraising and expressing emotions in self and others, regulating emotions in self and others, and using emotions in adaptive ways. Whereas trait EQ is usually defined as “a distinct and compound construct that lies at the lower levels of personality hierarchies” (Petrides et al., 2007, p. 283).

Theoretically, EQ, in general, is related to career success for two main reasons. First, individuals with higher EQ should also have higher inter-and intra-personal skills, which help them build interpersonal relationships with their co-workers and senior managers (Salami, 2008). As Jain (2012) stated, “EQ provides the protentional for performance, rather than performance itself” (p. 11). Indeed, studies have found that EQ or trait EQ is positively correlated with general success at work (Suhonen and Paasivaara, 2011; Amdurer et al., 2014; Jasielska, 2014; Latif et al., 2017). More specifically, in Amdurer et al.’s (2014) study, by studying 266 MBA graduates who had graduated between 5 and 19 years prior, the researchers found that trait EQ showed a long-term positive predictive influence on career satisfaction and success. Secondly, individuals with high EQ should be able to control and regulate their positive and negative emotional experiences at work which may transfer to organizational commitment. Studies have shown positive relationships between EQ and work success and organizational commitments (Petrides and Furnham, 2006; Taboli, 2013; Shafiq and Akram Rana, 2016; Baba, 2017). Specifically, in a study of 233 teachers at a higher educational institution in India, Baba (2017) found a positive significant relationship between trait EI and organizational commitment.

However, previous studies also reported some contradictory findings regarding the relationship between trait EQ and career success. For example, Latif et al. (2017) found that trait EQ had no direct effect on job performance for high school teachers. Similarly, researchers found that trait EQ had no direct or indirect effects on organizational commitment for 234 employees in an Iranian organization (Aghdasi et al., 2011).

### Adversity quotient

For career success, individuals also need to have the competency or capabilities to deal with the adversities and difficulties that they encountered in the workplace and daily life. In 1997, Stolz proposed the concept of AQ, describing it as a factor that can largely determine our success in work and life, more so than IQ or EQ. Even though Stoltz (1997) purposed AQ can predict various aspects of work, few empirical studies have focused on the topic of AQ. Among these limited studies, only one article was found that explored the positive relationship between AQ and career adaptability (Tian and Fan, 2014). Because of the limited empirical research on AQ as well as the critical role of resilience and persistence or grit in AQ (Stoltz, 1997; Suryaningrum et al., 2020), the current study purposed that individuals’ resilience and grit can be incorporated into the broader construct of AQ. In the current study, AQ was defined as the capacity to effectively cope and recover from internal and external obstacles, which may include perseverance and dynamic adaptation.

Werner and Smith (1982) carried out a longitudinal study of a community by following the same cohort (*N* = 700) for 30 years, starting in childhood, and found the significant role of resilience in helping children to do very well in later life, despite the risk factors that they faced during early childhood development. Studies found that individual resilience had a positive influence on their success at work (Tait, 2008; Wei and Taormina, 2014; Salisu et al., 2020), and even organizational success or change (e.g., Lengnick-Hall et al., 2011; Moenkemeyer et al., 2012; Cooper et al., 2013). Most of the previous studies did not explore the direct effect of resilience, particularly as a representation of AQ, on career success (e.g., Carstens et al., 2021; Guillén, 2021; Yu et al., 2022), and the effect of resilience on career success is mostly context-based (e.g., Fletcher and Sarkar, 2013).

Meanwhile, Duckworth et al. (2007) indicated grit involves “working strenuously toward challenges, maintaining effort and interest over years despite failure, adversity, and plateaus in progress”(p. 1087). They proposed that grit accounts for an average of 4% of the variance in success in life and career success. Concerning factors associated with career success, even though no studies have specifically examined the relationship between grit and work success, researchers have demonstrated a positive relationship between grit and academic success as well as retention in the military, workplace, school, and marriage (Eskreis-Winkler et al., 2014). Meanwhile, Hill et al. (2016) found that grit was positively correlated with purpose commitment (*r* = 0.44, *p* < 0.05) in a sample of 337 undergraduate students in Canada. However, there is no previous research on how grit affects organizational commitment. Grit, as another construct under the broader construct of AQ purposed in the current study, showed significant influence on individuals’ achievement-related domains but fewer studies focused on the career context (e.g., Clark and Plano Clark, 2019), and the effect on academic success was even challenged in Finland adolescents (Tang et al., 2019).

### The current study

The primary purpose of this study was to investigate the effects of trait EQ and AQ on career success, particularly in the domain of job position and organizational commitment, among Chinese adults. It was hypothesized that all aspects of trait EQ and AQ would have a positive influence on job position and organizational commitment.

## Methods

### Participants

In the current study, 256 participants were participated. Of those, 39.8% (102) were men and 60.2% (154) were women. The mean age was 36 years old (*SD* = 10.58). Most participants had earned a college degree (64.5%), were married (71.5%), and worked more than 40 h per week (61.3%). The working position was divided into three main categories (i.e., Tiers 1, 2, and 3). Tier 1 included entry-level positions that required little preparation regarding education, experience, and training to perform the work, such as assistants and sales associates. Tier 2 included positions that required some preparation regarding education, experience, and training to perform the work, such as grade school teachers and accountants. Tier 3 included the positions that required considerable preparation regarding education, experience, and training to perform the work, such as professors and managers. Participants indicated the highest position they had held, which was coded by the researchers into these three tiers. Participants currently working in cities were also divided into three tiers based on the city size. Tier 1 cities included major cities, such as Beijing, Shanghai, and Guangzhou. Tier 2 cities included the capital cities of each province in China, such as Shenyang and Urumqi. Tier 3 cities were those not included in Tiers 1 and 2. Cities were indicated by participants and coded by the researchers into these three tiers. More detailed demographic information is provided in Table 1.

**Table 1:** Total responses (*N*) and percentages for each demographic variable.

Variables	*N* (percentage)
Gender	256
Male	102 (*39.8%*)
Female	154 (*60.2%*)
Age	256
Age 18–34	128 (*50%*)
Age 35–50	98 (*38.3%*)
Age 51–69	30 (*11.7%*)
Age 70–87	0 (*0%*)
Education	256
Elementary and below	0 (*0%*)
Middle school	9 (*3.5%*)
High school	21 (*8.2%*)
University or college	165 (*64.5%*)
Masters	50 (*19.5%*)
PhD and above	11 (*4.3%*)
Marital Status	256
Married	183 (*71.5%*)
Divorce	11 (*4.3%*)
In a relationship	27 (*10.5%*)
Single	35 (*13.7%*)
Working status	256
Less than 40 h per week	99 (*38.7%*)
More than 40 h per week	157 (*61.3%*)
Highest working position	225
Tier 1	96 (*42.7%*)
Tier 2	73 (*32.4%*)
Tier 3	56 (*24.9%*)
City	256
Tier 1	14 (*5.5%*)
Tier 2	81 (*31.6%*)
Tier 3	161 (*62.9%*)

Highest working positions: Tier 1 positions required little education, experience, and training to perform the occupation; Tier 2 positions required some preparation; and Tier 3 positions required considerable preparation. City: Tier 1 included major cities (e.g., Beijing and Shanghai); Tier 2 included capital cities (e.g., Shenyang and Urumqi); and Tier 3 included cities not in to Tiers 1 and 2.

### Measures

#### Emotional quotient

The scale used to measure EQ was developed by Schutte et al. (1998), based on Salovey and Mayer’s (1990) model of EI. Schutte et al. (1998) developed the 33-item SREIT that comprises three factors: appraisal and expression of emotion (13 items), regulation of emotion (10 items), and utilization of emotion (10 items). Schutte et al. (1998) reported internal consistency reliability (*α* = 0.87) and test–retest reliability (*α* = 0.78), and the measurements were validated in China (Zhao et al., 2021). Respondents are asked to rate how well each statement describes them using a 5-point Likert scale (*1* = *strongly disagree* to *5* = *strongly agree*); however, in the current study, this was changed to a 4-point Likert scale, due to the mid-point effect or social desirability bias (Garland, 1991). Example questions for appraisal and expression of emotion include “emotions are one of the things that make my life worth living” and “I like to share my emotions with others.” Example questions for regulation of emotion include “I expect that I will do well on most things I try” and “other people find it easy to confide in me.” Example questions for utilization of emotion include “I expect good things to happen” and “I arrange events others enjoy.”

#### Adversity quotient

Adversity quotient was measured in two aspects, resilience and grit. Resilience was measured by using the RS-25 (Wagnild and Young, 1993). Grit was measured using the Grit Scale (Duckworth et al., 2007).

The RS-25 is a 25-item self-report measurement of resilience developed by Wagnild and Young (1993). In the original study, the researchers reported reliability of 0.91. Respondents are asked to indicate their agreement with each item on a 7-point Likert scale (*1* = *disagree* to *7* = *agree*). However, in the current study, this was changed to a 6-point Likert scale, due to the mid-point effect or social desirability bias (Garland, 1991). The measurement comprises two sub-factors: personal competence (17 items) and acceptance of self and life (8 items). Examples of questions for personal competence include “maintaining interest in things is important to me” and “I can be on my own if I have to be.” Example questions for acceptance of self and life include “I seldom wonder what the point of it all is” and “I take things 1 day at time.”

The Grit Scale is a 12-item self-report measurement of grit developed by Duckworth et al. (2007), who reported a range of reliabilities from 0.77 to 0.85. Respondents are asked to indicate how much each statement describes them, using a 5-point scale (*1* = *not like me at all*, *2* = *not much like me*, *3* = *somewhat like me*, *4* = *mostly like me*, and *5* = *very much like me*). The Grit Scale comprises two factors: consistency of interest (6 items) and perseverance of effort (6 items). All six items of the consistency of interest subscale were reverse-coded. Example questions for consistency of interest include “I become interested in new pursuits every few months” and “my interests change from year to year.” Example questions for the perseverance of effort include “I finish whatever I begin” and “setbacks do not discourage me.”

To assess the construct of AQ using RS-25 and GRIT scales, we performed factor and bifactor analyses. The results of the comparisons with different models of the structure of AQ were available from the corresponding author.

#### Organizational commitment

Organizational commitment was measured using the ACNCS (Allen and Meyer, 1990). The ACNCS is a 24-item self-report measurement that assesses organizational commitment in three aspects: affective commitment (AC; 8 items), continuance commitment (CC; 8 items), and normative commitment (NC; 8 items). According to Allen and Meyer’s (1990) three-component model, AC refers to employees’ psychological connection with an organization, CC refers to employees’ perceived viable alternatives available in the job market, and NC refers to employees’ perceived experiences associated with an organization. In the original study, reliability for the AC, CC, and NC subscales was 0.87, 0.75, and 0.79, respectively. Respondents are asked to indicate how much they agree with each item using a 7-point Likert scale (*1* = *disagree* to *7* = *strongly agree*); however, in the current study, a 6-point Likert scale was used in which the mid-point was deleted, due to the mid-point effect or social desirability bias (Garland, 1991). Example questions for AC include “I would be very happy to spend the rest of my career with this organization” and “I enjoy discussing my organization with people outside of it.” Example questions for CC include “I am not afraid of what might happen if I quit my job without having another one lined up” and “it would be very hard for me to leave my organization right now, even if I wanted to.” Example questions for NC include “I think that people these days move from company to company too often” and “I do not believe that a person must always be loyal to his or her organization.” Nine items of the ACNCS were reverse coded (i.e., four items on the AC scale, two items on the CC scale, and three items on the NC scale).

#### Basic demographic information

The present study collected basic demographic information including gender, age, city, education level, marital status, working status, and highest working position, which was used to represent objective career success.

### Procedure

Since the current study was conducted in China, all instruments were first translated into Chinese by the researcher and proofread by another person, who is fluent in both Chinese and English. Then all Chinese versions of the questionnaires went through the back-translation procedure (Brislin, 1986). After approval was received from the appropriate IRB, the questionnaire was administrated *via* WenJuanXing, which is an online survey platform similar to Qualtrics or SurveyMonkey. A QR code and a URL link were generated for the survey and put together in a poster distributed online through a social networking platform in China. Informed consent was collected before participants began the survey. All data in the current study were collected online. The questionnaire was open to the public for a month.

All participants were recruited online *via* social media. The snowball sampling method was used in the current study. All participants were encouraged to forward the poster to their social media. A total of 378 participants were recruited online. From that group, 122 participants were removed from the current study, as they did not meet the pre-requisite of being employed (either part-time or full-time). Therefore, 256 participants were totally involved in the current study, and they all being employed.

### Data analysis

Factor analysis was first performed for each questionnaire to examine its internal structure (e.g., confirmatory factor analysis). Internal reliability for each scale was determined by Cronbach’s (1951) alpha coefficients. Meanwhile, bi-factor analysis was performed to test the construct structure of AQ. Preliminary analyses of all scales were performed (e.g., mean, standard deviation (*SD*), and skewness). To identify the effect of specific aspects of EQ and AQ on job position and three aspects of organizational commitment, multiple regression analysis was conducted using SPSS version 23. Four analyses were performed independently regarding different aspects of career success, which were three types of organizational commitment and job position.

## Results

### Measurements validity and reliability

Based on the results from confirmatory factor analysis for the SREIT, three factors were confirmed. Two items were deleted due to low factor loadings, which left 31 items in total. Therefore, in the current study, the appraisal and expression of emotion subscale had 11 items, the regulation of emotion subscale had 10 items, and the utilization of emotion subscale had 10 items. Subscales were created by averaging all scores for each scale, which ranged from 1 to 4. Higher scores indicated a higher perceived emotional ability in that domain. Reliability for appraisal and expression of emotion, regulation of emotion, and utilization of emotion were 0.80, 0.79, and 0.76, respectively.

Based on the confirmatory factor analysis results for the RS-25, two subscales were confirmed as it in the original study. However, based on the factor loadings in the current study, the personal competence subscale had 9 items rather than 17 items, and the acceptance of self and life subscale had 16 items rather than 8 items in the original study. Subscales were created by averaging all scores in that subscale, which ranged from 1 to 6. Higher scores indicate higher levels of resilience for that specific domain. In the current study, the reliability for the personal competence and acceptance of self and life subscales were 0.87 and 0.93, respectively.

Based on the confirmatory factor analysis results for the Grit Scale, the same two subscales structure was found as in the original study. Subscales were created by averaging all scores in each subscale, ranging from 1 to 5. Higher scores indicate a higher level of grit. In the current study, the reliabilities for consistency of interest and perseverance of effort subscales were 0.81 and 0.83, respectively.

Finally, confirmatory factor analyses were performed for each of the three ACNCS subscales that were used to measure organizational commitment, which found the same internal structure was found as in the original study. However, items were deleted due to low factor loadings in each subscale. Two items were deleted from the AC subscale, one item was deleted from the CC and the NC subscale. Thus, in the current study, the AC, CC, and NC subscales contained 6, 7, and 7 items, respectively. Three subscales were created by averaging all scores for each subscale, which ranged from 1 to 6. Higher scores indicate higher levels of AC, CC, and NC. Reliability for the AC, CC, and NC subscales were 0.78, 0.90, and 0.80, respectively.

### Descriptive statistics

Table 2 presents the number of responses (*N*), means (*M*), standard deviations (*SD*), and skewness for study variables. Participants in the current study demonstrated medium to high levels of EQ in all three domains, ranging from 2.85 to 2.97. Similar to EQ, both the grit and resilience aspects of AQ showed medium or medium to high levels of AQ. Regarding aspects of organizational commitment, participants showed a medium commitment level for all three aspects (ranging from 3.54 to 3.98) with a large SD (ranging from 0.94 to 1.14). Finally, regarding the correlations among study variables, all variables are significant and positively correlated with each other, except for consistent of interest from AQ. Consistency of interest was significantly and negatively correlated to EQ and organizational commitment and showed a non-significant correlation with girt.

**Table 2:** Descriptive statistics and correlations between study variables.

Variable	1	2	3	4	5	6	7	8	9	10
*Emotional quotient (EQ) predictor*										
1. Appraisal and expression of emotion	-									
2. Regulation of emotion	0.70^**^	-								
3. Utilization of emotion	0.71^**^	0.75^**^	-							
*Adversity quotient (AQ) predictor*										
4. Resilience-Personal competence	0.62^**^	0.70^**^	0.61^**^	-						
5. Resilience-acceptance of self and life	0.62^**^	0.71^**^	0.59^**^	0.75^**^	-					
6. Grit-consistency of interest	−0.32^**^	−0.14^*^	−0.22^**^	−0.22^**^	−0.12	-				
7. Grit-perseverance of effort	0.51^**^	0.51^**^	0.49^**^	0.59^**^	0.68^**^	−0.12	-			
*Dependent variable*										
8. OC-affective commitment	0.32^**^	0.38^**^	0.35^**^	0.31^**^	0.36^**^	−0.15^**^	0.28^**^	-		
9. OC-continuance commitment	0.14^*^	0.19^**^	0.18^**^	0.26^**^	0.12	−0.15^*^	0.05	0.49^**^	-	
10. OC-normative commitment	0.27^**^	0.35^**^	0.27^**^	0.30^**^	0.35^**^	−0.13^*^	0.28^**^	0.62^**^	0.51^**^	-
*M*	2.85	2.96	2.97	4.77	4.39	3.18	3.45	3.54	3.98	3.69
SD	0.40	0.38	0.38	0.66	0.80	0.86	0.84	1.01	1.14	0.94
*a*	0.80	0.79	0.76	0.87	0.93	0.81	0.83	0.79	0.90	0.80

^*^p < 0.005. ^***^p < 0.001 (2-tailed).

OC: organizational commitment.

### Emotional quotient, adversity quotient, and organizational commitment

As shown in Table 3, three components of organizational commitment were involved in the current study. Allen and Meyer (1990) indicated that these three components were conceptually different, and each component was developed independently. Considering the psychometric structure of the ACNCS, three independent regression analyses were conducted.

**Table 3:** Regression coefficients in predicting organizational commitment and job position from emotional quotient (EQ) and adversity quotient (AQ) variables.

	Organizational commitment			Job position
	Affective commitment	Continuance commitment	Normative commitment	
	*Standardized b coefficient* (*SE*)	*Standardized b coefficient* (*SE*)	*Standardized b coefficient* (*SE*)	*Standardized b coefficient* (*SE*)
*EQ variables*				
Appraisal and expression of emotion	−0.02	−0.15	−0.01	0.07
	(0.19)	(0.20)	(0.16)	
Regulation of emotion	0.27^*^	0.07	0.15	−0.11
	(0.22)	(0.23)	(0.19)	
Utilization of emotion	−0.09	0.21^*^	−0.17	−0.12
	(0.19)	(0.21)	(0.17)	
*AQ variables*				
Grit-consistency of interest	0.17^*^	−0.07	−0.03	0.05
	(0.06)	(0.06)	(0.05)	
Grit-perseverance of effort	0.15	−0.13	0.17^*^	−0.06
	(0.08)	(0.08)	(0.07)	
RS-personal competence	−0.13	0.32^*^	−0.26^*^	0.05
	(0.12)	(0.12)	(0.10)	
RS-acceptance of self and life	0.18	−0.09	0.36^*^	0.39^*^
	(0.17)	(0.11)	(0.09)	

^*^p < 0.05. RS: resilience.

Variables that reached significant levels are bolded.

For AC, all three aspects of EQ (e.g., appraisal and expression of emotion, regulation of emotion, and utilization of emotion) and all four aspects of AQ (e.g., consistency of interest, perseverance of effort, personal competence, and acceptance of self and life) were used to predict AC. The overall predictive model was statistically significant, *F*(7, 256) = 6.39, *p* < 0.001, and accounted for approximately 15.3% of the variance for AC (*R*^2^ = 0.153, Adjusted *R*^2^ = 0.129). However, only one factor of EQ (regulation of emotion) and one factor of AQ (consistency of interest) showed a significant and positive regression effect on AC.

For CC, the same EQ and AQ variables were used to predict CC. Overall, the prediction model was statistically significant, *F*(7, 256) = 3.05, *p* = 0.05, and accounted for approximately 7.9% of the variance for CC (*R*^2^ = 0.079, Adjusted *R*^2^ = 0.053). Results further indicated that two out of the seven factors reached statistically significant levels. More specifically, the utilization of emotion positively predicted CC, and personal competence from the resilience aspect of AQ showed a positive regression weight in predicting CC.

For NC, the same EQ and AQ variables were again used to predict NC. Overall, the prediction model was statistically significant, *F*(7, 256) = 5.62, *p* < 0.001, and accounted for approximately 13.7% of the variance for NC (*R^2^* = 0.137, Adjusted *R^2^* = 0.113). Results further indicated that three out of seven factors reached statistically significant levels. More specifically, perseverance of effort and acceptance of self and life showed significant positive regression weights in predicting NC, whereas personal competence showed a significant negative effect on NC.

### Emotional quotient, adversity quotient, and job position

Regarding the relationship between EQ, AQ, and job position, all three aspects of EQ and four aspects of AQ were used to predict job position. Overall, the prediction model did not reach statistically significant, *F*(7, 256) = 3.49, *p* = 0.001, and accounted for approximately 10.1% of the variance for the job position (*R*^2^ = 0.101, Adjusted *R*^2^ = 0.072). Regression analysis results indicated that only one out of seven factors in the model significantly predicated job position, which was the acceptance of self and life from the resilience aspect of AQ.

## Discussion

Following Shockley et al.’s (2016) recommendation that individual career success should consider both OCS and SCS, the current study used job position as a proxy for OCS and self-reported perceived organizational commitment as a proxy for SCS. By exploring the relationship between different aspects of trait EQ and AQ and organizational commitment and job position, the current study revealed several significant findings.

### Emotional quotient, adversity quotient, and organizational commitment

The positive relationship between trait EQ and organizational commitment was partially supported. In the present study, rather than viewing organizational commitment as a single construct and exploring how it was affected by personal competence (e.g., Bruning and Snyder, 1983), the organizational commitment was examined in three different aspects, namely AC, NC, and CC. Although the results of the present study conflicted with those of Shafiq and Akram Rana (2016), who found trait EQ was a significant and positive predictor of all three components of organizational commitment (i.e., AC, CC, and NC), the results of the present study indicated that only specific aspect of trait EQ had a positive influence on organizational commitment. Specifically, the present study revealed that individuals’ ability to regulate their emotions positively predicted AC. In other words, individuals with higher abilities to regulate their own and others’ emotions are more likely to become psychologically connected and committed to an organization. A possible explanation for this finding is that when an individual has strong abilities to regulate their emotions and those of others, they are more likely to develop strong emotional or psychological connections with their co-workers. Such overall strong emotional connections further ensure individuals will build strong affective attachments to an organization. Individuals willing to be more committed to their organization can increase their ability to regulate their emotions and those of others, in which they will be able to better deal with their working relationship and get psychologically connected to their organization. Moreover, the utilization of emotion positively predicted CC. Utilization of emotion refers to “flexible planning, creative thinking, redirected attention and motivation” (Schutte et al., 1998, p. 168), and CC refers to individuals’ perception of the cost of leaving an organization. Thus, individuals who usually have flexible plans and active thoughts are less likely to leave the organization because they have a better ability to balance the cost-effectiveness between staying and leaving the organization. In the other words, individuals with a higher ability to utilize emotions might be more rational rather than emotional, which helped them rationally analyze the cost of leaving an organization. It might be important for organizations, especially human resources personnel, to pay attention to such individuals since they might also be the individuals that suddenly leave the organization as soon as they find better work on the job market. Future studies might also consider exploring the relationship between the utilization of emotion, turnover, and continuous commitment to provide empirical evidence on the above assumption.

The positive relationship between AQ and organizational commitment was complicated. Four factors of AQ (i.e., consistency of interest, perseverance of effort, personal competence, and acceptance of self and life) were found to independently and positively predict different aspect(s) of organizational commitment. Specifically, consistency of interest was found to positively predict AC but not CC or NC. Considering the demographic characteristics of the sample in the current study (e.g., the average age was 36), as well as the culture in China, most individuals may not be sure of what their interests are. Interest has been defined as “a person’s relatively enduring predisposition to reengage particular content over time” (Hidi and Renninger, 2006, p. 113). It is possible that there is a bidirectional influence between consistency of interest and work. The more time and energy individuals spend at their jobs, the more likely their jobs are to become their interest. Therefore, due to the enduring predisposition and continuing re-engagement with job-related activities (i.e., consistency of interest), individuals become more emotionally and psychologically involved, connected, and eventually affectively committed to an organization. The possible bidirectional influence between consistency of interest and affective commitment shed the light on the fact that interest in the job position might not be a necessary consideration when recruiting new employees. Therefore, when recruiting potential employees, human resources personnel should prioritize employees’ talent and qualifications rather than their personal interest in the position.

Perseverance of effort and acceptance of self and life were found to positively predict NC. In the other words, individuals who are characterized as having more perseverance for their efforts or goals and have a more adaptive, balanced, and flexible perspective of life (i.e., acceptance of self and life) tend to show more obligation, loyalty, and responsibility to an organization (i.e., NC). One previous study found that more adaptable individuals, especially with career-related adaptive behaviors (i.e., career planning), displayed more loyalty to an organization during an organizational restructuring period (Klehe et al., 2011). Beyond the positive effects of adaptability (i.e., acceptance of self and life), the present study also revealed the positive influence of the internal quality of perseverance (i.e., perseverance of effort) on organizational loyalty (i.e., NC) for in-service employees. During recruiting and new-employee training period, human resource personnel might consider examining and cultivating individuals’ acceptance of self and life and perseverance. In such ways, individuals might be more loyal and committed to their organization.

A notable finding was that one aspect of AQ (i.e., personal competence) positively predicted CC, but negatively predicted NC. As described by Meyer and Allen (1991), CC is developed when individuals invest a lot in an organization and perceive a lack of alternative opportunities in the job market. NC is affected by experiences (i.e., loyalty-related) individuals have both before and after they start work with an organization. Therefore, results from the current study revealed that individuals who are characterized as having “self-reliance, independence, determination, invincibility, mastery, resourcefulness, and perseverance” (i.e., personal competence; Wagnild and Young, 1993, p. 174) may also invest a lot into an organization (i.e., CC), but demonstrate less loyalty (i.e., NC). By analyzing 124 published studies, a previous meta-analysis found that perceived personal competence was considered to be an antecedent of organizational commitment (Mathieu and Zajac, 1990). Building on this previous study, the current study provided more empirical evidence on the positive effect of personal competence on CC and the negative effect on NC. This might shed light that individuals with strong personal competence are a double-edged sword. Even though they can be the employees that have the highest productivity, they can also be the ones who are most likely to leave the organization if they have bad experiences at work. Therefore, organizational management and human resources personnel should pay extra attention to the working experience of individuals with strong personal competence and productivity. They might consider providing timely psychological and material support to such individuals to ensure they have a good experience with the organization.

### Emotional quotient, adversity quotient, and job position

The hypothesized positive relationship between trait EQ and job position was rejected. No significant and positive relationship was found between the three aspects of trait EQ and job position, which contradicted previous research findings. For example, Sultana et al. (2016) found that EQ positively predicted bank employees’ OCS (i.e., salary) in Pakistan. Moreover, another study found that trait EQ was a significant predictor of job performance beyond the effect of IQ among research and developmental scientists in China (Law et al., 2008) and Malaysian administrators (Jorfi et al., 2012). Thus, it might suggest that even if EQ can positively influence individuals’ job performance, it might not necessarily help them to achieve higher positions. Previous studies indicated the importance of one of the important Chinese cultural characteristics of *guanxi* relationships, which can be understood as ‘a network of personally defined reciprocal bonds’ (Redding et al., 1994, p. 656), on organization positions (Farh et al., 1998; Tsang, 1998; Chen and Francesco, 2000). These contradictory findings indicated that more research is needed on the relationship between trait EQ and OCS, especially in the context of China.

The positive relationship between AQ and job position was partially proved. Specifically, only one aspect of AQ (i.e., acceptance of self and life) positively predicted job position. In other words, individuals who have “adaptability, balance, flexibility, and a balanced perspective of life” (i.e., acceptance of self and life; Wagnild and Young, 1993, p. 175) are usually able to reach a higher position within their organizations compared to those without these characteristics. One possible explanation of this relationship is that, as discussed earlier, people with high levels of self-or life-acceptance tend to have higher loyalty and responsibility (i.e., NC) toward an organization. Such high levels of loyalty and responsibility could help individuals become more successful at their jobs, which in turn could help them reach higher job positions. Therefore, if individuals want to reach a higher position in their work, they might consider increasing their AQ, especially cultivating the ability to accept and balance their self and life.

### Contributions and limitations

Aside from the above discussion, several implications can be drawn from the current study. First, numerous researchers have investigated the potential factors that contribute to individuals’ career success. Aside from the well-accepted effect of cognitive ability (i.e., IQ) on career success, the current study provides insight into the significant influence of specific types of trait EQ and AQ on both OCS and SCS. For organizational professionals, when considering increasing an organization’s overall productivity or employees’ commitment toward an organization, it is important to cultivate employees’ EQ and AQ, especially their emotional regulation abilities and personal qualities (i.e., perseverance, stable interests, and acceptance of self and life). Meanwhile, human resources professionals might want to pay attention to individuals who have a higher ability to utilize their emotions since they are more likely to see alternative working opportunities in the job market. For individuals, when considering career success, it is important to cultivate EQ-and AQ-related skills. Secondly, most previous studies considered EQ and AQ as a single construct when studying the predictive power of factors related to one’s career success (e.g., Mendoza and Hontiveros, 2017; Urquijo et al., 2019). The current study explored, in detail, how specific types of trait EQ and AQ can facilitate individuals to become more successful in their jobs. Thirdly, because of the brief history of AQ and limited research on the topic, the current research provided more empirical evidence regarding its influence on individual career success. Fourth, rather than assessing the commonly used variables to represent individuals’ OCS and SCS (i.e., job or career satisfaction, salary, and promotion), the current study employed job position and organizational commitment to represent individuals’ OCS and SCS, respectively, and found significant effects of EQ and AQ on them. The results lend support to the possibility of using organizational commitment to represent SCS. More specifically, the findings of the current research highlight the important role of the organization and its effect on individuals’ perception of career success. Future researchers might want to explore this subject in greater detail and provide additional empirical evidence. Finally, most of the previous research on this topic focused on Western cultures; however, the current study, after carefully examining the psychometric structure of the measurements used, provided empirical evidence of the effects of trait EQ and AQ on career success in Eastern culture.

The present study has several limitations that must be overcome in future research. On the one hand, since the participants were recruited in China, some specific aspects of Chinese culture need to be considered. For example, individuals in China are praised more when they are calmer and show few emotions in public settings. When dealing with relationships, especially in the workplace, it is considered wiser to hide or control one’s actual emotions, rather than express them. Therefore, one aspect of EQ (i.e., appraisal and expression of emotion) showed no significant predictive power for both organizational commitment and job position. On the other hand, all measurements were developed based on Western cultures, and the content and values embedded in the measurements might not be suitable for the culture and values in China. Even though all measurements were validated in the current study, future studies need to consider the differences between individualism and collectivism.

## Data availability statement

The raw data supporting the conclusions of this article will be made available by the authors, without undue reservation.

## Ethics statement

The studies involving human participants were reviewed and approved by University of Missouri-Saint Louis. The patients/participants provided their written informed consent to participate in this study.

## Author contributions

YZ was the corresponding author of the paper. BS contributed to the final approval of the version to be published. All authors contributed to the article and approved the submitted version.

## Conflict of interest

The authors declare that the research was conducted in the absence of any commercial or financial relationships that could be construed as a potential conflict of interest.

## Publisher’s note

All claims expressed in this article are solely those of the authors and do not necessarily represent those of their affiliated organizations, or those of the publisher, the editors and the reviewers. Any product that may be evaluated in this article, or claim that may be made by its manufacturer, is not guaranteed or endorsed by the publisher.
